# Practical Chemistry and Pharmacy

**Published:** 1895-11

**Authors:** 

**Affiliations:** LaGrange, Ga.


### PRACTICAL CHEMISTRY AND PHARMACY.

EDITED BY HENRY R. SLACK, M.D., PH.M., LAGRANGE, GA.

### DETECTION OF BLOOD-STAINS IN MEDICO-LEGAL CASES.

The detection and demonstration of blood spots on rusty iron is made more difficult by the fact that the iron renders it impossible to obtain heman crystals. F. Gautier states that in such cases hydrogen peroxide furnishes a certain test of the absence of blood. The slightest trace of blood, on being wet with a solution of hydrogen peroxide, instantaneously releases oxygen, the minute bubbles of which may be seen with the unaided eye. These bubbles collect on the surface of the drop, which presently, under a magnifying glass, appears quite foamy with them, and looking white, even to the unaided eye. The technique is very simple. A portion of the suspected blood substance—a slight scraping is sufficient—is placed on a glass slip, the back of which is covered with black paper. Cover the suspected material with a drop of weakly alkaline water, and let stand for a few minutes, or until the substance is softened, and then add a drop of peroxide of hydrogen solution. If there is the slightest trace of blood present, the globules of oxygen instantaneously appear, and they are so characteristic that any confusion of them with air bubbles is impossible. If the reaction does not occur, it is certain demonstration of the absence of blood. It is to be added that the appearance of the reaction is not a demonstration that blood is present, as other animal liquids behave in the same manner.—*Zeit. für Analy. Chemic.*

### Test for Tubercle Bacilli.

Of the various methods employed for staining tubercle bacilli in sputum, Bemysek recommends the following: The sputum to be examined is evenly divided and pressed between two sterilized object glasses, and then exposed to the air, preferably under a bell-jar, to dry. All heat is to be avoided, as the stains then become less distinct. The dry sputum is now moistened with a mixture composed of a concentrated alcoholic solution of fuchsine 4 parts, carbolic acid 5 parts, and water 45 parts, and gently warmed over a spirit lamp until vapors rise. It is then washed with water and stained with a soluion of methylene blue, to which 10per cent. of sulphuric acid is added. After four to six minutes it is again washed with water, and finally dried. Through this treatment the tubercle bacilli acquire a dark red color, while the rest of the specimen is colored light blue. Other bacteria are not stained by this procedure. Very good results are also said to be obtained by staining with an alkaline solution of methylene blue and malachite green; but this is a slower process than the above.—*Pharm. Ztz.* XL., p. 481.

### Quantitative Estimation of Sugar in Urine.

M. Cregny describes a simple method for obtaining approximately the quantity of sugar in diabetic urine, which, he suggests, would be of convenience to practitioners residing in remote districts where the necessary chemical appliances for analysis are not always available. The following is the *modus operandi*, which is a simplification of M. Duhomme's process: 2 cc. each of soda solution, sp. gr. 1.330, and Fehling's solution are poured into a burette and the urine to be examined is added drop by drop, and to stop the moment the blue coloration has disappeared. The amount of sugar present is calculated according to a prepared chart by the number of drops required to produce reduction of the reagent. The tabulation is as follows: 4 drops of urine reducing 2 cc. of Fehling's solution represents 50 grams of sugar per liter; 8 drops of urine reducing 2 cc. of Fehling's solution represents 25 grams of sugar per liter; 10 drops of urine reducing 2 cc. of Fehling's solution represents 20 grams of sugar per liter, etc.—*Drug. Cir. and Chem. Gaz.*

---

### CLEANING RUSTY INSTRUMENTS.

Broder gives the following as an effective method of cleaning rusty instruments. Fill a suitable vessel with a saturated solution of chloride of tin in distilled water, immerse the rusty instruments and let them remain over night. Rub dry with chamois after rinsing in running water, and they will be of a bright silvery whiteness.—*Jour. Brit. Dent. Asso.*

